# Pregnancy and lactation-associated osteoporosis in a postpartum woman with twin pregnancy: a 5-year follow-up case report

**DOI:** 10.3389/fendo.2026.1931693

**Published:** 2026-08-26

**Authors:** Hussain Muthasim Adnan, Xiaoyun Gao, Xiuqi Yu, Chunyu Zhang

**Affiliations:** Department of Geriatrics, The Second Affiliated Hospital of Dalian Medical University, Dalian, Liaoning, China

**Keywords:** bone mineral density, postpartum back pain, pregnancy and lactation-associated osteoporosis, twin pregnancy, vertebral compression fractures, zoledronic acid

## Abstract

**Introduction:**

Pregnancy and lactation-associated osteoporosis (PLO) is a rare but potentially disabling metabolic bone disorder that typically emerges in late pregnancy or the early postpartum period. Because its symptoms often present as common postpartum musculoskeletal complaints, diagnosis is frequently delayed until vertebral fractures are detected. This case report describes a postpartum woman with a twin pregnancy who developed multiple vertebral compression fractures, illustrating the diagnostic challenges, clinical features, and long-term outcomes of PLO.

**Case presentation:**

A 32-year-old woman presented with persistent low back pain two weeks after cesarean delivery of twins. She had been exclusively breastfeeding and reported inadequate dietary intake and recent weight loss. Thoracolumbar MRI revealed acute compression fractures at T12, L1, and L5. Laboratory evaluation demonstrated low 25-hydroxyvitamin D and parathyroid hormone levels, markedly elevated β-CTX and P1NP, and elevated prolactin. DXA showed significantly reduced lumbar bone mineral density (Z-score −3.25). Clinically significant secondary causes of osteoporosis were excluded based on available testing, and PLO was diagnosed. She was treated with calcium carbonate, calcitriol, alfacalcidol, calcitonin, and intravenous zoledronic acid (initial 5 mg dose during admission, with a second 5 mg dose administered at 1-year follow-up). Following treatment, biochemical markers normalized. At follow-up, MRI confirmed fracture healing, DXA demonstrated improved bone mineral density, and the patient reported complete resolution of symptoms. Long-term evaluation at 5 years showed sustained clinical stability and continued improvement in BMD.

**Conclusion:**

This case highlights the importance of considering PLO in postpartum women with severe back pain, particularly in the presence of risk factors such as low BMI, inadequate calcium intake, vitamin D insufficiency, exclusive breastfeeding, and twin pregnancy. Early recognition and management can prevent prolonged morbidity and support continued improvement in bone health.

## Introduction

Pregnancy and lactation-associated osteoporosis (PLO) is a rare metabolic bone disorder characterized by reduced bone mineral density and an increased risk of fragility fractures during late pregnancy or the early postpartum period (1). The condition is frequently underdiagnosed because its symptoms, most commonly severe low back pain, overlap with common postpartum musculoskeletal complaints, leading many women to experience delays in appropriate evaluation and treatment (2, 3). Although the true incidence remains uncertain, estimates suggest that PLO affects approximately 0.4 per 100,000 women, underscoring its rarity and the ongoing challenges in recognition (1, 4). More recent population-based studies, however, report slightly higher rates, approximately 4–8 cases per 100,000 pregnancies, particularly when vertebral fractures during lactation are included, suggesting that PLO may be under-recognized in clinical practice (5).

The pathophysiology of PLO is multifactorial and incompletely understood. During pregnancy, intestinal calcium absorption increases to meet fetal skeletal demands, whereas lactation induces a shift toward skeletal resorption mediated largely by parathyroid hormone-related protein (PTHrP) (4, 6). Additional contributors may include inadequate calcium or vitamin D intake, low pre-pregnancy bone mass, endocrine abnormalities, and lifestyle factors such as low BMI or prolonged breastfeeding (1, 3). Twin or multiple gestations further intensify maternal calcium requirements, potentially accelerating bone loss and increasing fracture risk (3). Emerging evidence also suggests a genetic predisposition, with variants in LRP5 and WNT1 implicated in impaired bone formation and more severe clinical presentations (7).

Despite growing awareness, standardized diagnostic and therapeutic guidelines for PLO remain lacking. Diagnosis typically relies on a combination of clinical suspicion, bone mineral density assessment via DXA, and imaging, most commonly MRI, to identify vertebral fractures (3). Management strategies vary widely and may include calcium and vitamin D supplementation, cessation of breastfeeding, antiresorptive therapy, and physical rehabilitation (8, 9).

## Case presentation

**Figure 1:**
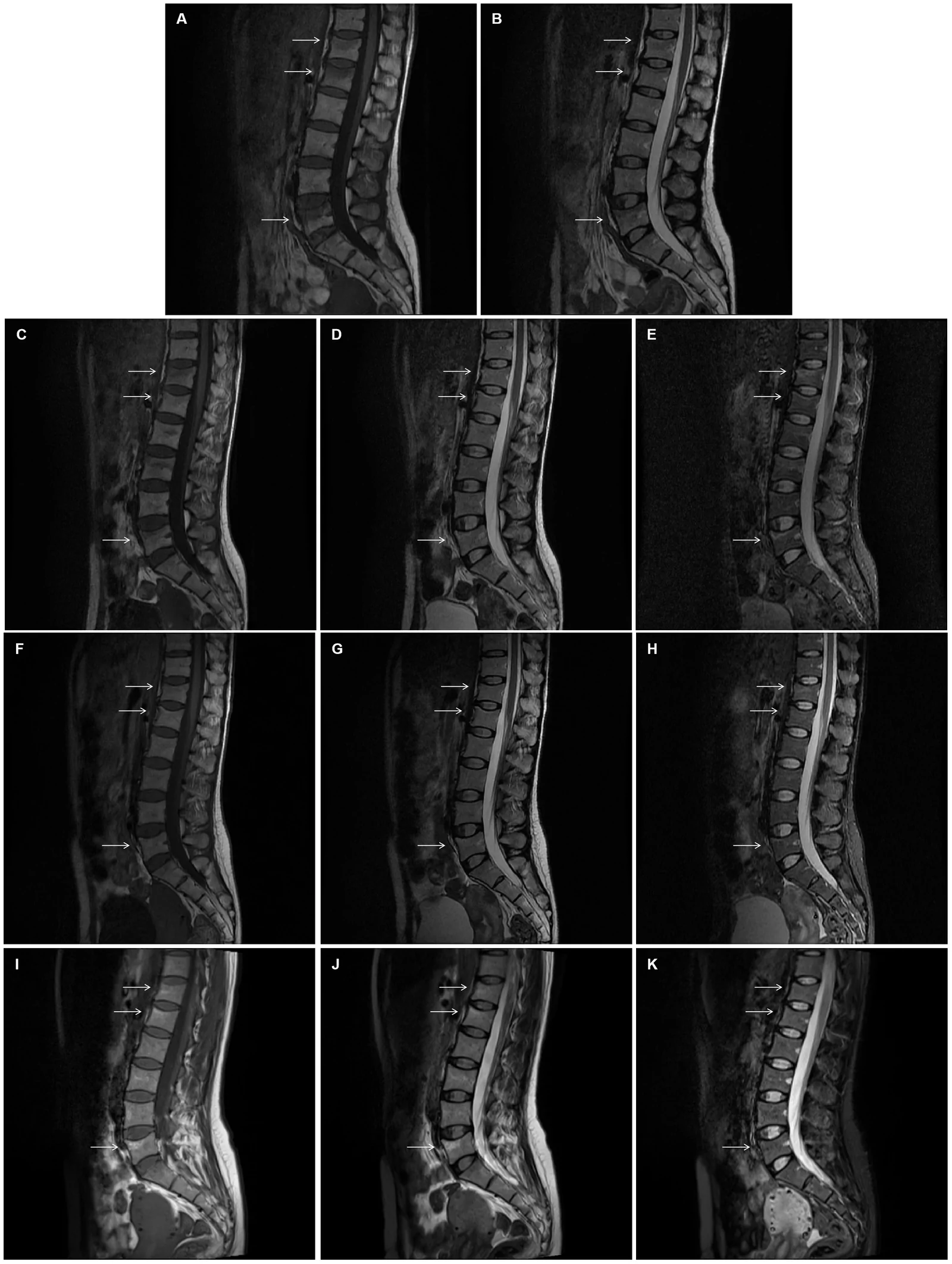
Early, post-treatment, and follow-up MRI of the thoracolumbar spine. **(A)** Sagittal T1-weighted MRI from June 6, 2021. **(B)** Sagittal T2-weighted MRI from June 6, 2021. **(C)** Sagittal T1-weighted MRI from July 2, 2021. **(D)** Sagittal T2-weighted MRI from July 2, 2021. **(E)** Sagittal STIR sequence from July 2, 2021. **(F)** Sagittal T1-weighted MRI from July 21, 2021. **(G)** Sagittal T2-weighted MRI from July 21, 2021. **(H)** Sagittal STIR sequence from July 21, 2021. **(I)** Sagittal T1-weighted MRI from March 3, 2022. **(J)** Sagittal T2-weighted MRI from March 3, 2022. **(K)** Sagittal STIR sequence from March 3, 2022.

A 32-year-old female patient presented to the spinal surgery clinic of the Second Affiliated Hospital of Dalian Medical University, Dalian City, Liaoning, China, on June 4, 2021. She presented with the chief complaint of low back pain for 2 weeks. Prior to this hospital visit, she was treated at a different hospital, with bandaging and many sessions of acupuncture, massage, and other treatments. She did, however, mention recent weight loss compared with her prenatal time. She experienced no relief with these treatments and presented to the Spinal Surgery Clinic of the hospital. An MRI thoracolumbar spine was performed on June 6, 2021 and revealed compression fractures in T12, L1 and L5 vertebrae (**Figures 1A, B**). She was diagnosed as a case of “spinal fracture”. She was treated with symptomatic pain relief treatment and was advised bed rest. Following this visit, she had multiple visits but with no relief.

Three days prior to admission, her lower back pain worsened and was accompanied by discomfort in the right hip, prompting her to return to the hospital on July 2, 2021. She initially presented to the orthopedic department and then was referred by the orthopedic surgeon to the Osteoporosis Clinic of the Department of Geriatrics, Second Affiliated Hospital of Dalian Medical University. The patient developed low back pain after bending down to pick up her child at home, around one and a half months back at the time of her admission. The low back pain was aggravated with weight bearing. She had no history of fall or trauma and there was no history of inactivity or limb numbness following the onset of low back pain. Her postpartum diet was light, mostly millet porridge and green vegetables. The patient reported vomiting during the first three months of pregnancy and diarrhea for approximately one month after delivery which may have added to her impaired nutritional status. She has a history of appendectomy 10 years ago; otherwise, her past history was unremarkable as she denied any history of any other diseases. Family history was negative for osteoporosis or fragility fractures in her mother. A review of her obstetric history reveals, she underwent cesarean section on March 18, 2021 resulting in live twin female infants, and started exclusive breastfeeding after surgery. Her daily breast milk production averaged 1200 mL. The patient continued exclusive breastfeeding until her admission on July 2, 2021, despite persistent back pain and MRI-confirmed vertebral fractures on June 6, 2021. This delay likely reflects limited awareness of PLO, as postpartum back pain is often attributed to benign musculoskeletal strain. Continued lactation may have contributed to ongoing skeletal calcium loss and worsening symptoms prior to diagnosis.

Repeat MRI thoracolumbar spine done on July 2, 2021 also showed vertebral compression fractures in T12, L1 and L5 vertebrae (**Figures 1C–E**). She was admitted as a suspected case of Pregnancy and Lactation-associated osteoporosis in the inpatient ward of the Department of Geriatrics for further testing.

At the time of admission, she was vitally stable (T: 36 °C, P: 78 beats/min, R: 14 breaths/min, BP: 110/82mmHg). Her height was measured at 155 cm and weight was measured to be 43 kg during the post partum checkup. Therefore her BMI was calculated at 17.89 kg/m^2^. She was lying in bed, with obvious pain in the lower back when turning over. There were no abnormalities in muscle strength, muscle tone, and deep and superficial sensation in the limbs were intact. Physical examination of her cardiovascular system, respiratory system, abdominal examination revealed no abnormalities.

On initial laboratory investigations done at admission, serum calcium levels were 2.51mmol/L and found to be within the normal range. However her serum phosphorous levels were slightly elevated at 1.78mmol/L. Her 25-hydroxyvitamin D levels were also found to be low at 17.38ng/mL and PTH levels were also low at 3.75pg/mL. She was found to have an elevated prolactin level at 1959.38μIU/mL. Her Procollagen Type 1 N-terminal propeptide (P1NP) was also found to be elevated at 133.3ng/mL and beta-C-terminal telopeptide (β-CTX) was also markedly elevated at 2465pg/mL. All other routine blood tests done showed no abnormal results and were all within the normal ranges (**Table 1**).

**Table 1:** Summary of laboratory parameters and bone mineral density values at admission and during follow-up (2021–2026).

Investigation	On admission (07/03/2021)	At discharge (07/24/2021)	Follow-up (03/03/2022)	5 year follow-up (06/11/2026)	Reference range
Serum Calcium (mmol/L)	2.51	2.15	2.44	2.34	2.11-2.52
Serum Phosphorous (mmol/L)	1.78	1.47	1.27	1.15	0.85-1.51
25-hydroxyvitamin D (ng/mL)	17.38	–	31.05	16.40	30-100
PTH (pg/mL)	3.75	–	29.07	36.70	15-65
Prolactin (μIU/mL)	1959.38	–	–	–	59-619
Procollagen Type 1 N-Terminal propeptide (P1NP) (ng/mL)	133.3	–	–	22.70	Female: Premenopausal 15.13-58.59; Postmenopausal 20.25-76.31
beta-C-terminal telopeptide (β-CTX) (pg/mL)	2465	–	–	210	Female: Premenopausal 299-573; Postmenopausal 566–1008
DXA Scan Z score	-3.25	–	-2.64	-1.40	
DXA Scan BMD (g/cm²)	0.6207	–	0.7504	0.764	

On July 6, 2021, a routine DXA scan was done to ascertain her BMD. Bone densitometry of the 2^nd^-4^th^ lumbar vertebrae, femoral neck and total hip showed lowest Z value of -3.25 and the bone mineral density value of 0.6207g/cm² (2^nd^-4^th^ lumbar vertebrae).

A broad differential including osteomalacia, traumatic fracture, and infectious spondylitis was excluded based on the patient’s biochemical profile (low PTH), absence of trauma, and lack of fever or inflammatory signs. Her endocrine work-up, including thyroid function and antibodies, cortisol, ACTH, adrenal catecholamines, progesterone, testosterone, estradiol, LH, and FSH was entirely normal, eliminating common hormonal contributors. Routine blood, urine, and stool tests, along with normal renal function, electrolytes, and tumor markers, showed no signs of hematologic disease or malignancy. Autoimmune and connective tissue disorders were excluded through negative rheumatologic immune markers. Ultrasound scans were done to rule out all abdominal, hepatobiliary, pancreatic, urological, thyroid, breast, uterine adnexal, and lower limb venous abnormalities. Whole body bone imaging showed no clear tumor-related abnormal bone focus. These findings excluded clinically significant secondary causes based on available testing, supporting the diagnosis of pregnancy and lactation-associated osteoporosis.

**Figure 2:**
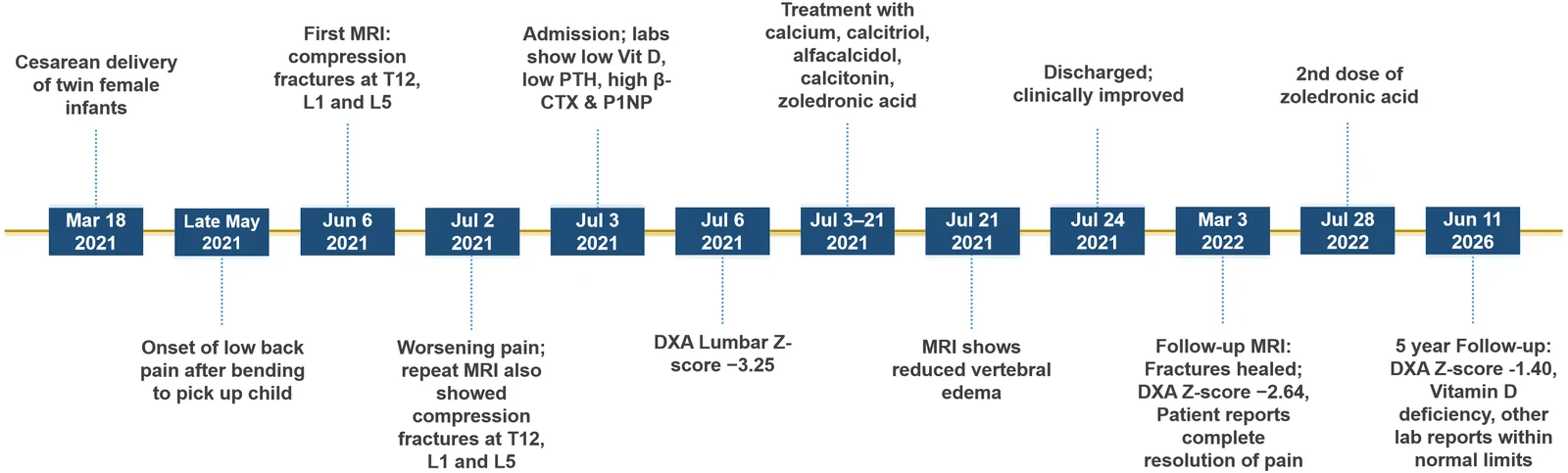
Timeline of clinical events.

Based on the above findings, the final diagnosis was made as compression fracture of the 12^th^ thoracic, 1^st^ lumbar and 5^th^ lumbar vertebrae secondary to pregnancy lactation-associated osteoporosis (**Figure 2**).

**Figure 3:**
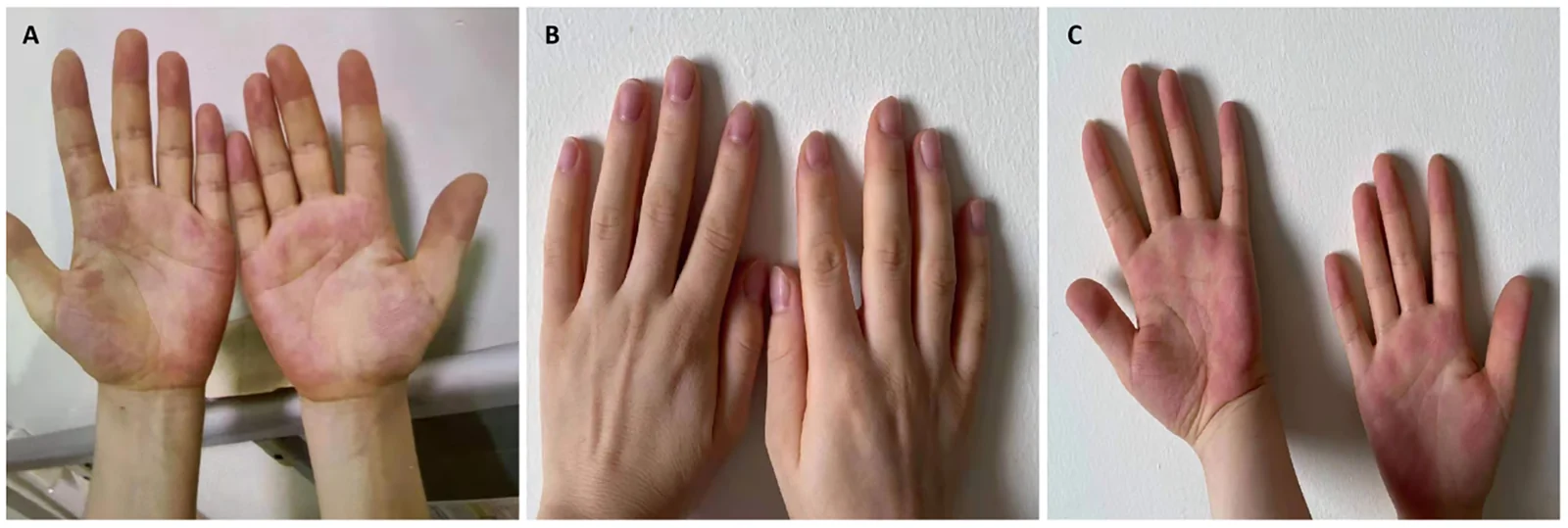
Allergic reaction to calcitonin. Panel **(A)** (July 16, 2021) shows initial palmar erythema shortly after calcitonin administration. Panels **(B, C)** (July 18, 2021) show a more extensive erythematous reaction involving both dorsal and palmar surfaces, indicating worsening hypersensitivity.

After her admission on July 3, she was started on calcium carbonate 1.5 g once daily and calcitriol 0.5 μg once daily. Breastfeeding was discontinued at the time of admission to reduce further skeletal calcium loss and support early recovery. On July 6, calcium carbonate was increased to 1.5 g twice daily (3 g/day) while continuing calcitriol 0.5 μg once daily. On July 8, when her serum calcium rose above the normal range, calcitriol was stopped, calcium carbonate was reduced back to 1.5 g once daily, and alfacalcidol 0.25 μg once daily was added. This regimen (calcium carbonate 1.5 g once daily plus alfacalcidol 0.25 μg once daily) was given for 5 days. Subsequently, calcitonin 100 IU once daily subcutaneously was started on July 14. On July 16, on the 3rd day after starting calcitonin, the patient developed a worsening erythematous reaction and the drug was immediately discontinued. The reaction progressed over the following days, with worsening erythema documented on July 18 (**Figure 3**). Finally, she received a single intravenous dose of zoledronic acid 5 mg on July 16. During this period, her serum calcium increased transiently to a peak of 2.91 mmol/L (reference 2.11–2.52 mmol/L), which resolved after dose adjustment and did not require specific intervention.

After this treatment, prior to her discharge, MRI of thoracolumbar region was repeated on July 21, 2021. (**Figures 1F–H**) It showed markedly reduced edematous changes in T12, L1 and L5 vertebrae, slightly herniated disc in L5/S1. Both serum calcium and phosphorous levels returned to normal after the Intravenous Zoledronic acid and was within normal levels at the time of her discharge from the hospital on July 24, 2021.

The patient had a follow-up visit on March 3, 2022. An MRI was done during this follow-up visit and it showed that the vertebral fractures seen in the previous imaging have healed (**Figures 1I–K**). The laboratory reports from the follow-up showed all parameters within normal limits and her DXA scan also shows improvement with a Z-score of -2.64 and an improved bone mineral density value of 0.7504g/cm².

At her follow-up visit on March 3, 2022, the patient reported complete resolution of her low back pain and no longer experienced any discomfort. She was able to resume normal daily activities, including caring for her children, without any limitations. The patient received a second intravenous dose of zoledronic acid 5 mg on July 28, 2022 during routine outpatient follow-up. At that time, she reported no back pain or functional limitations as well. The decision to administer a second dose of zoledronic acid was based on clinical judgment and the patient’s recovery trajectory. Although her symptoms had resolved, her bone mineral density remained below the expected range for age, and a second infusion was given to consolidate skeletal gains and reduce the risk of future fractures.

The patient returned for a follow-up on June 11, 2026. The patient remained clinically stable and reported no recurrence of low back pain or functional limitations. Laboratory investigations at this visit showed serum calcium, phosphorus, PTH, P1NP, and β-CTX levels all within their respective normal ranges. However, her 25-hydroxyvitamin D level was again found to be low at 16.40 ng/mL. At the 5-year follow-up, the patient was no longer taking calcium or vitamin D supplements regularly. This may partially explain the recurrence of vitamin D insufficiency despite otherwise stable biochemical parameters and improved bone mineral density. A repeat DXA scan demonstrated continued improvement in bone mineral density, with a lumbar Z-score of –1.40 and a BMD of 0.764 g/cm², indicating substantial recovery compared with her initial presentation. Overall, the findings from this follow-up visit confirmed long-term clinical stability and sustained improvement in bone health.

## Discussion

Pregnancy and lactation-associated osteoporosis (PLO) is an uncommon but clinically significant cause of severe postpartum back pain. Because symptoms often overlap with benign musculoskeletal complaints, diagnosis is frequently delayed until vertebral fractures are identified on imaging. The thoracolumbar spine is the most commonly affected region, consistent with the fracture distribution observed in this patient at T12, L1, and L5, which aligns with prior reports describing vertebral fragility as the hallmark of PLO (2, 3). More recent analyses also emphasize that the majority of PLO-related fractures occur during lactation rather than pregnancy, reflecting the period of greatest skeletal vulnerability (5).

The pathophysiology of PLO is multifactorial. During pregnancy, intestinal calcium absorption increases to meet fetal skeletal demands, but lactation shifts maternal calcium homeostasis toward skeletal resorption mediated by parathyroid hormone-related protein (PTHrP) (4, 6). Suppressed parathyroid hormone and low vitamin D levels, as seen in this patient, further impair calcium balance and may exacerbate bone loss. Twin pregnancy imposes an even greater calcium requirement, accelerating maternal skeletal depletion. This pattern of suppressed PTH with elevated bone turnover markers is consistent with the lactation-driven physiology described in prior studies, where PTHrP-mediated skeletal resorption becomes the dominant mechanism of calcium mobilization during breastfeeding (4, 6). Recent mechanistic work has also highlighted the roles of oxytocin, sympathetic nervous system activation, and bone marrow adipose tissue lipolysis in amplifying lactation-related bone resorption, offering additional insight into why some women experience excessive skeletal loss during this period (10). This is supported by recent literature indicating that multigestational pregnancies substantially increase maternal calcium mobilization and may predispose to more severe clinical manifestations of PLO (3).

During normal lactation, women who exclusively breastfeed lose 5-10% of lumbar spine BMD over 3–6 months with full recovery usually occurring within 6–12 months after weaning (3). These reversible changes reflect physiologic adaptations, including elevated prolactin, suppressed PTH, and increased bone turnover markers, and do not cause symptoms or fractures (3).

In this case, the abnormalities exceeded these expected ranges. Her β-CTX level (2465 pg/mL) was markedly elevated, her lumbar spine Z-score (−3.25) was substantially below the degree of bone loss linked to normal lactation. She developed multiple vertebral compression fractures which is a finding that does not occur in normal lactation physiology. This pathologic exaggeration of normal lactation physiology which is consistent with PLO was likely amplified by her twin pregnancy which can double or triple maternal calcium losses (3) as well as her low BMI, vitamin D insufficiency and inadequate dietary intake.

Several risk factors in this case, including low BMI, limited dietary calcium intake, vitamin D insufficiency, and exclusive breastfeeding, are well-recognized contributors to PLO (1, 3). The patient’s breast milk production of approximately 1200 mL per day was also higher than the average production reported of a singleton pregnancy (11). Her low BMI is particularly meaningful, as women with reduced peak bone mass and smaller skeletal reserves are more vulnerable to the metabolic demands of pregnancy and lactation. Beyond these clinical factors, recent studies have drawn attention to a genetic component. Variants in genes such as LRP5 and WNT1 have been linked to impaired bone formation and appear more frequently in women with severe or recurrent PLO, suggesting that a subset of patients may carry an underlying hereditary predisposition (7). When these clinical and genetic vulnerabilities converge, the usual skeletal adaptations of pregnancy and lactation may no longer be sufficient, placing susceptible women at heightened risk for accelerated bone loss and fragility fractures.

MRI played a central role in both diagnosis and follow-up. The patient’s initial scans demonstrated acute vertebral compression fractures accompanied by marked marrow edema, while subsequent imaging showed a steady reduction in edema and eventual fracture healing. This radiological progression closely reflects the recovery pattern described in the literature, where marrow edema typically resolves over several months after breastfeeding is discontinued and appropriate therapy is initiated (8).

Management of PLO remains non-standardized due to its rarity, and current therapeutic approaches generally aim to correct calcium and vitamin D deficiency, reduce excessive bone resorption, and support bone formation. In this patient, sequential treatment with calcium, calcitriol, alfacalcidol, calcitonin, and ultimately zoledronic acid was associated with normalization of biochemical markers, clear radiological improvement, and complete clinical recovery. At the 5-year follow-up, her 25-hydroxyvitamin D level remained low (16.40 ng/mL), indicating persistent insufficiency despite otherwise stable biochemical parameters. Although long-term postpartum vitamin D trajectories are not well described in the literature, low vitamin D status is a well-recognized contributor to impaired skeletal recovery and increased susceptibility to metabolic bone stress during pregnancy and lactation (1, 4).

Calcium, vitamin D analogs (calcitriol and alfacalcidol), and calcitonin were given in succession to address vitamin D deficiency and provide short-term pain relief for her vertebral fractures. Correction of calcium and vitamin D deficiency before initiating antiresorptive therapy is recommended to optimize treatment response and minimize the risk of hypocalcemia during bisphosphonate administration (4, 6). Calcitonin may also provide short-term analgesic benefit in acute vertebral fractures, offering symptomatic relief while longer-acting therapies take effect (8, 12). Zoledronic acid was selected because alternative parenteral agents (denosumab and teriparatide) were unavailable at the time, its once a year dose offered better patient compliance. The patient was counseled regarding risks and future pregnancy planning prior to treatment. The second dose of zoledronic acid was administered at the 1-year follow-up after the repeat DXA scan showed persistent BMD below expectations (Z-score -2.64, BMD 0.7504 g/cm²). Zoledronic acid, a potent intravenous bisphosphonate, has demonstrated particular efficacy in rapidly reducing bone turnover and facilitating vertebral healing in PLO (8). The observed improvements cannot be attributed to zoledronic acid alone. Calcium repletion, vitamin D analogs, breastfeeding cessation, and calcitonin were all used concurrently, and each may have contributed to the clinical recovery.

Most published PLO cases report follow-up durations of 6–18 months, with limited data describing long-term skeletal outcomes beyond two years (1, 8). The 5-year follow-up in this case provides valuable insight into the sustained recovery trajectory of BMD and bone turnover markers, demonstrating that substantial improvement can continue for several years after the initial event. Although bone mineral density typically returns to baseline after weaning, recent evidence suggests that microarchitectural changes may persist despite normalization of BMD, indicating that recovery may be more complex than previously appreciated (10).

This case report has several limitations. Pre-pregnancy laboratory values and BMD were not available. PTHrP was not measured, and formal genetic testing for LRP5/WNT1 was not performed. Quantitative dietary calcium intake was not assessed. Furthermore, the single-case design limits generalizability of the findings.

## Patient perspective

After giving birth, my back pain kept getting worse, and I became very worried. When the doctors explained the diagnosis to me, I finally understood the real reason, and my heart felt more at ease. After starting the treatment, my pain improved quickly, and I was able to return to taking care of my children again.

## Conclusion

Pregnancy and lactation-associated osteoporosis is an uncommon and overlooked cause of severe postpartum back pain. This case shows the need to consider PLO in postpartum women; particularly those with identifiable risk factors such as low BMI, inadequate calcium intake, vitamin D insufficiency, exclusive breastfeeding, and twin pregnancy.

In this patient, prompt diagnosis and treatment were associated with normalization of biochemical markers, radiological healing of vertebral fractures, improvement in bone mineral density, and complete resolution of symptoms. Increased awareness among clinicians may help reduce diagnostic delays, prevent prolonged morbidity, and improve outcomes for affected women.

## Data Availability

The original contributions presented in the study are included in the article/**Supplementary Material**. Further inquiries can be directed to the corresponding author.

## References

[1] Scioscia MF Zanchetta MB . Recent insights into pregnancy and lactation-associated osteoporosis (PLO). Int J Womens Health. (2023) 15:1227–38. doi: 10.2147/IJWH.S366254 37551335 PMC10404404

[2] Miles B Panchbhavi M Mackey JD . Analysis of fracture incidence in 135 patients with pregnancy and lactation osteoporosis (PLO). Cureus. (2021) 13:e19011. doi: 10.7759/cureus.19011 34824928 PMC8610434

[3] Kovacs CS . Maternal mineral and bone metabolism during pregnancy, lactation, and post-weaning recovery. Physiol Rev. (2016) 96:449–547. doi: 10.1152/physrev.00027.2015 26887676

[4] Carsote M Turturea MR Valea A Buescu C Nistor C Turturea IF . Bridging the gap: Pregnancy-and lactation-associated osteoporosis. Diagnostics (Basel). (2023) 13:1615. doi: 10.3390/diagnostics13091615 37175006 PMC10177839

[5] Kovacs CS . Pregnancy and lactation associated bone fragility. J Endocr Soc. (2025) 9:bvaf126. doi: 10.1210/jendso/bvaf126 40837111 PMC12362253

[6] Kovacs CS Feingold KR Adler RA Ahmed SF Anawalt B Blackman MR . Calcium and phosphate metabolism and related disorders during pregnancy and lactation. In: Feingold KR Adler RA Ahmed SF , editors. Endotext. MDText.com, Inc, South Dartmouth (MA (2024). [Internet]. doi: 10.1016/j.ecl.2011.08.002

[7] Butscheidt S Tsourdi E Rolvien T Delsmann A Stürznickel J Barvencik F . Relevant genetic variants are common in women with pregnancy and lactation-associated osteoporosis (PLO) and predispose to more severe clinical manifestations. Bone. (2021) 147:115911. doi: 10.1016/j.bone.2021.115911 33716164

[8] Zhang M Chen P Li B Du J Pan T Chen J . Approach to the patient with pregnancy and lactation-associated osteoporosis: A case report and a review of the literature. Med (Baltimore). (2017) 96:e8671. doi: 10.1097/MD.0000000000008671 29145296 PMC5704841

[9] Sahni P Edeer AO Lindsay R . Rehabilitation of pregnancy and lactation-associated osteoporosis and vertebral fractures: A case report. HSS J. (2024) 20:298–305. doi: 10.1177/15563316231167148 39281988 PMC11393619

[10] Winter EM Ireland A Butterfield NC Haffner-Luntzer M Horcajada MN Veldhuis-Vlug AG . Pregnancy and lactation, a challenge for the skeleton. Endocr Connect. (2020) 9:R143–57. doi: 10.1530/EC-20-0055 32438342 PMC7354730

[11] Geddes DT Gridneva Z Perrella SL Mitoulas LR Kent JC Stinson LF . 25 years of research in human lactation: From discovery to translation. Nutrients. (2021) 13:3071. doi: 10.3390/nu13093071 34578947 PMC8465002

[12] Yazdani J Khiavi RK Ghavimi MA Mortazavi A Hagh EJ Ahmadpour F . Calcitonina como agente analgésico: revisão dos mecanismos de ação e das aplicações clínicas [Calcitonin as an analgesic agent: review of mechanisms of action and clinical applications. Braz J Anesthesiol. (2019) 69:594–604. doi: 10.1016/j.bjan.2019.08.004 31810524 PMC9391842

