# Supplementary material for: Pregnancy and lactation-associated osteoporosis in a postpartum woman with twin pregnancy: a 5-year follow-up case report
Source: Front Endocrinol (Lausanne). 2026 Aug 26;17:1931693. doi: 10.3389/fendo.2026.1931693 (PMC13553373; doi:10.3389/fendo.2026.1931693)
Supplement: Supplementary file 1 [file DataSheet1.pdf]

# CARE Checklist of information to include when writing a case report

CARE case report guidelines

(cc) BY-NC-ND

| Topic | Item | Checklist item description | Reported on Line |
|---|---|---|---|
| Title | 1 | The diagnosis or intervention of primary focus followed by the words "case report" | Title page |
| Key Words | 2 | 2 to 5 key words that identify diagnoses or interventions in this case report, including "case report" | Keywords section |
| Abstract (no references) | 3a | Introduction: What is unique about this case and what does it add to the scientific literature? | Abstract – Introduction |
| | 3b | Main symptoms and/or important clinical findings | Abstract – Case Presentation |
| | 3c | The main diagnoses, therapeutic interventions, and outcomes | Abstract – Case Presentation |
| | 3d | Conclusion—What is the main "take-away" lesson(s) from this case? | Abstract – Conclusion |
| Introduction | 4 | One or two paragraphs summarizing why this case is unique (**may include references**) | Introduction section |
| Patient Information | 5a | De-identified patient specific information. | Case Presentation – first paragraph |
| | 5b | Primary concerns and symptoms of the patient. | Case Presentation – "low back pain…" |
| | 5c | Medical, family, and psycho-social history including relevant genetic information | Case Presentation – obstetric history, diet, past surgery |
| | 5d | Relevant past interventions with outcomes | Case Presentation – acupuncture, massage, bandaging |
| Clinical Findings | 6 | Describe significant physical examination (PE) and important clinical findings. | Case Presentation – physical exam paragraph |
| Timeline | 7 | Historical and current information from this episode of care organized as a timeline | Figure 2 (Timeline of Clinical Events) |
| Diagnostic Assessment | 8a | Diagnostic testing (such as PE, laboratory testing, imaging, surveys). | Case Presentation – MRI, labs, DXA |
| | 8b | Diagnostic challenges (such as access to testing, financial, or cultural) | Case Presentation – "delay likely reflects limited awareness" |
| | 8c | Diagnosis (including other diagnoses considered) | Case Presentation – exclusion of secondary causes |
| | 8d | Prognosis (such as staging in oncology) where applicable | Not applicable |
| Therapeutic Intervention | 9a | Types of therapeutic intervention (such as pharmacologic, surgical, preventive, self-care) | Case Presentation – treatment section |
| | 9b | Administration of therapeutic intervention (such as dosage, strength, duration) | Case Presentation – detailed dosing July 3–21 |
| | 9c | Changes in therapeutic intervention (with rationale) | Case Presentation – calcitriol stopped, alfacalcidol added, calcitonin discontinued |
| Follow-up and Outcomes | 10a | Clinician and patient-assessed outcomes (if available) | Case Presentation – follow-up 2022 & 2026 |
| | 10b | Important follow-up diagnostic and other test results | MRI 2022, DXA 2022 & 2026, labs |
| | 10c | Intervention adherence and tolerability (How was this assessed?) | Case Presentation – calcitonin hypersensitivity |
| | 10d | Adverse and unanticipated events | Figure 3 – allergic reaction |
| Discussion | 11a | A scientific discussion of the strengths AND limitations associated with this case report | Discussion – first paragraph |
| | 11b | Discussion of the relevant medical literature **with references**. | Discussion – multiple references |
| | 11c | The scientific rationale for any conclusions (including assessment of possible causes) | Discussion – pathophysiology, risk factors, treatment logic |
| | 11d | The primary "take-away" lessons of this case report (without references) in a one paragraph conclusion | Conclusion section |
| Patient Perspective | 12 | The patient should share their perspective in one to two paragraphs on the treatment(s) they received | Patient Perspective section |
| Informed Consent | 13 | Did the patient give informed consent? Please provide if requested | Yes ☑ No ☐ |
